# Hard Study

**Published:** 1858-07

**Authors:** 


### HARD STUDY.

It is a very general mistake, that hard study kills people. It is often written in obituary notices, especially of young men who have died at college, that they were the victims of hard study—kind of martyrs to literature, and to their anxiety to enter on the more active and responsible duties of life. None but a weak-minded ignoramus would write such twaddle as this. Only give the brain seven hours of regular and undisturbed repose out of every twenty-four, and it will be invigorated by all the activities that can be imposed upon it, if the person will only eat plain nourishing food, at three regular times each day, and spend two or three hours of daylight in active exercise on foot or horseback. No instance can be found, in all history or biography, where, under such circumstances, any amount of brain-work has even been productive of serious bodily inconvenience. On the contrary, brain-work is a positive pleasure to thinking men—it is literally their meat and drink, a pure delight, a labor which brings no weariness in half a century's duration; as living instances, there are, Prince METTERNICH, HUMBOLDT, PALMERSTON, and on our own side of the water, Dr. NOTT, and others, all approaching their nineties; and of the great dead, ADAMS, and BENTON, and CLAY, and CALHOUN, and CHARLES CALDWELL, all of whose minds worked with seeming undiminished vigor to the close of a long life. Away, then, with the impertinent falsity, "*He died of hard study.*" "*He died of animal indulgence,*" as no brute beast dies; and the fact of possessing a high intellect, and made higher by cultivation, only adds enormity to the crime of reckless, inconsiderate self-destruction. The fashion of the times is to send a common drunkard, neck and heels, to perdition; while the young gluttons, who shorten their lives half a century by gourmandizing three times every day, are almost saintified. There is, possibly, some shadow of excuse for a winebibber; for, according to report, he and his company are made joyous for half a night, with hours of sound sleep afterwards, to boot. But the miserable gourmand eats his meal in silence; all his joy is during its brief passage down the throat, and, within an hour, his groans begin, to continue for the four or five hours following, until the half-corrupted mass is passed off from his laboring stomach, during which time, if you were to listen to him, he would make you believe he had all the plagues of Egypt and of Job, with Job's wife thrown in—the only mercy, by the way, which wasn't doubled!

A gentleman writes: "With a most vigorous constitution, tested by twenty-five years of hard toil as a student and teacher, never kept from my business a day by sickness, and never under a doctor's care an hour, I am earnest to do what I can for the physical, as well as the moral and intellectual health of my generation." This man, with others like him, as BENTON, ADAMS, NOTT, and HUMBOLDT, who had moral courage and intelligence enough to live temperately, and rationally, keeping the animal appetites in subjection, these men live long and study hard to the last hour of life, almost; and all who follow their high example of systematic temperance, may do likewise, and make the world feel for good, the impress of their lives, instead of having their light go out, in the obscurity of an early grave, through their lust for animal gratifications.

In high bodily health, brain-work, like body-work, gives an appetite; and if that appetite is only indulged regularly and moderately, any student may live to a good old age, with an hour or two of judicious exercise out of doors, every day; and, in the end, save years of efficient labor by it.

